# Developing and Applying a Typology of ‘Better for You’ Claims on Alcohol Products

**DOI:** 10.1111/dar.70163

**Published:** 2026-05-01

**Authors:** Asad Yusoff, Simone Pettigrew, Bella Sträuli, Paula O'Brien, Jacqueline Bowden, Mark Petticrew, Alexandra Jones

**Affiliations:** ^1^ The George Institute for Global Health Sydney Australia; ^2^ Griffith Health School of Medicine and Dentistry Griffith University Gold Coast Australia; ^3^ Melbourne Law School The University of Melbourne Melbourne Australia; ^4^ National Centre for Education and Training on Addiction Flinders University Adelaide Australia; ^5^ Faculty of Public Health and Policy London School of Hygiene and Tropical Medicine London UK

**Keywords:** alcohol, claims, labelling, prevention

## Abstract

**Introduction:**

To appeal to health‐conscious consumers, alcohol producers are marketing products with a range of ‘better for you’ claims. This study aims to: (i) develop a typology of text‐based ‘better for you’ claims used on alcohol products; (ii) assess the prevalence of these claims in the Australian market overall and by alcohol category; and (iii) identify the extent to which products display multiple claims.

**Methods:**

Between March and November 2024, images of 5982 unique alcohol products were captured in‐store from three retailers in Australia. The products were coded for presence and type of ‘better for you’ claims, with each claim classified into an emergent typology of categories. Descriptive analyses were conducted overall and by alcohol category.

**Results:**

Eight major categories of claims were identified: nutrient content, energy content, absence of ingredient, low alcohol, fruit/botanical references, natural, processing and vegan claims. Across the sample, 35% of products displayed at least one claim (range: 1–9 claims), and one in eight displayed multiple claims. Cider (91%) and premix (75%) categories contained the highest proportion of products featuring at least one claim.

**Discussion and Conclusions:**

The use of claims across the Australian alcohol market appears to be quite common, particularly on premix and cider products. Limited regulation of claims allows companies to portray products as ‘better for you’, potentially misleading consumers and taking advantage of their efforts to be health‐conscious. Future work in Australia should prioritise developing policies that restrict the use of claims on alcohol products, particularly those shown to be misleading.

## 1 Introduction

Alcohol consumption continues to present a major public health challenge in many countries. It is estimated that alcohol is responsible for 4.7% of the global burden of disease and contributes to 2.6 million deaths per year [1]. It has proven difficult to address alcohol‐related harms due to alcohol use being deeply ingrained in many cultures [2]. However, over the last decade, there has been some progress with per capita consumption declining in several demographic groups in many high‐income nations [3]. Apparent drivers for this decline include a reduction in drinking among younger age groups, the rise of the ‘sober curious’ movement, an overall increase in health consciousness, and greater public awareness of the risks associated with alcohol use [4, 5, 6, 7].

One way the alcohol industry has responded to shifting consumer preferences is by marketing products using a range of on‐pack text‐based claims to convey the idea that these alcohol products (defined as > 1.15% alcohol by volume [ABV]) are ‘better for you’ [8, 9]. These ‘better for you’ claims (hereafter ‘claims’) attempt to appeal to health‐conscious consumers by highlighting a variety of product attributes including ingredients (e.g., botanical flavours), nutrient content (e.g., low carbohydrate content) and manufacturing practices (e.g., organic) [9, 10]. In addition to objective product features, claims can also make references to vague health‐related descriptors such as ‘natural’ and ‘fresh’ [11].

A key concern surrounding the use of claims is their potential to influence how consumers view and use alcoholic products. Nutrient content claims, for example, have been shown to increase the perceived healthiness of alcohol products and reduce perceived harm in comparison to standard versions of the product [12, 13]. The misperception of product healthiness caused by claims (also known as a health halo effect) may be particularly dangerous for alcohol products given the potential to: (i) distract consumers from the health risks associated with even low levels of consumption [14, 15]; and (ii) imply to consumers that alcohol can be good for you or have health benefits.

At present there is limited evidence on the use of claims in the international context [16]. In the Australian market, however, previous studies investigating the prevalence of nutrition and health‐related claims on alcohol products have found that beer and premix products (also known as ready‐to‐drink beverages) typically have a high prevalence of such claims, and that ‘natural’ claims are the most common [17, 18, 19]. A recent study analysed the use of ‘virtue’ claims (claims that are health‐related, eco‐related and/or relating to social causes) on new alcoholic products that entered the Australian market between 2013 and 2023. The use of virtue claims was found to have increased significantly over the study period, which was predominately attributed to an increase in health‐oriented claims [11].

The Australia New Zealand Food Standards Code (‘the Code’) restricts on‐pack claims for alcoholic beverages (over 1.15% ABV) by prohibiting ‘health claims’ (defined as a claim that ‘states, suggests or implies that a food or a property of food has, or may have, a health effect’ [20], such as ‘Calcium for healthy bones and teeth’ [21]). The Code also only allows specific nutrition content claims related to energy content, gluten, sugar and carbohydrates [22]. Claims are prohibited about other aspects of the nutrition content of alcohol products, such as their fat content. There are also many types of information that can be used to make claims but are not regulated under the Code, such as the organic origin of the ingredients used to create the product. In addition to the rules under the Code, producers of alcohol products must comply with general fair trading laws, including the Australian Consumer Law under the Competition and Consumer Act 2010 (‘the Act’), which prohibits false, misleading, or deceptive representations being made to consumers in respect of goods [23]. This most basic of consumer protections extends to claims made on alcohol products.

Due to the rapidly evolving nature of the alcohol product market [24], there is a need for up‐to‐date, detailed data on the full range of claims being used by the industry on different products. Additionally, to facilitate consistency in the monitoring and cross‐country comparison of claims on alcohol products, the development of a classification system is required. Previous audits of the alcohol market have identified a range of claims; however, no studies appear to have synthesised the variety of claims used by the industry into a comprehensive typology. It should be noted that while useful guides, existing typologies and labelling frameworks developed for claims on packaged food may not be fit‐for‐purpose to analyse alcohol products for two reasons. First, typologies of claims on food products predominantly focus on health and structure/function claims that are not allowed on alcohol products in most nations. Second, because alcohol is a psychoactive, toxic substance, a different approach to analysing and regulating claims on alcohol is needed [25]. To address these research gaps, the aims of this study were to: (i) develop a typology of text‐based ‘better for you’ claims for alcohol products; (ii) assess the frequency of use of these claims overall and by alcohol product category; and (iii) identify the extent to which alcohol products display multiple ‘better for you’ claims.

## 2 Methods

Data were collected between March and November 2024 from Dan Murphy's, BWS and ALDI stores, representing three major Australian alcohol retailers that make up approximately 60% of the Australian off‐trade alcohol supply [26, 27]. All stores were located in Sydney, Australia, with each store selected on the basis of: (i) being within a 5‐km radius of the Central Business District; and (ii) store managers providing permissions for data collectors to enter the premises. Data collection was conducted using the established Alcohol Industry Monitoring System (AIMS), which is based on the FoodSwitch infrastructure and methodology [28]. AIMS monitors the products available for sale in the Australian market via annual in‐store collections. Trained data collectors used a bespoke smartphone application to capture images of all sides of every unique product (excluding products in locked cabinets). After collection, a trained data coder extracted all on‐pack information from the images following a comprehensive codebook. Coding reliability was checked by a second coder and automated checks for potential anomalies (e.g., ABV% that exceeds the expected level for a particular alcohol category). Information captured in the AIMS database includes, but is not limited to, product name, alcohol category, alcohol content and any on‐pack claims.

Alcoholic products (defined as > 1.15% ABV) were classified into one of five categories: beer, wine, spirits, premix and cider. To accurately reflect the number of labels present in the market, different‐sized versions of the same product (e.g., single vs. multi‐pack) were treated as distinct products, whereas duplicate products of identical size and packaging sourced from different stores were included only once in the dataset. Consistent with information available to consumers at the point of purchase, only text from the outermost layer of multi‐pack variations was extracted from the product images (e.g., for a 4‐pack of beer cans, text from the outer cardboard packaging was included rather than information on each individual can).

In line with previous work examining the marketing of unhealthy beverages, a ‘better for you’ claim was defined as any text on packaging (excluding the nutrition information panel and ingredients list) “that either claims or implies that a product has health‐related benefits or is a healthier option” [10]. This included cases where a claim was present in a product name (e.g., Hahn Zero Carb Lager) and, building on previous work on alcohol products [11, 16, 17, 19], references to fruit or fruit flavours. Fruit terms were classified as a ‘better for you’ claim for two reasons. First, the use of fruit flavours on harmful products, such as vapes, has been shown to lead consumers (in particular those in younger age groups) to believe products featuring such claims are less harmful [29, 30]. Given alcohol's known health harms, it was deemed that the use of fruit claims on alcohol products likely has a similar effect. Second, consumers may be misled about the fruit content of an alcohol product because there is no standard about how much fruit content must be included in an alcoholic beverage for the product to make a claim about ‘fruit’ (e.g., Passionfruit Vodka). There is also no requirement for an ingredients list on alcoholic beverages sold in Australia (as compared to food), such that consumers have no means of informing themselves about the fruit content of the alcoholic beverage [22].

The typology developed in this study was informed by ideal‐type analysis, an approach commonly used to systematically generate typologies from qualitative data [31]. First, a single coder (AY) assessed all individual claims in the dataset and grouped them according to similar subject matters (e.g., low sugar and sugar‐free would be grouped as ‘sugar claims’). The grouped claims were then compared against one another resulting in the clustering of claims into an emergent set of typology categories that were mutually exclusive and comprehensively exhaustive of the various ways claims could refer to a product's ‘better for you’ characteristics. The results were subsequently reviewed and refined by the broader authorship team to finalise the typology.

All statistical analysis was conducted using Stata 16 [32]. First, relative frequencies were calculated to determine the proportion of products featuring at least one claim, multiple claims, and each of the categories identified during the typological analysis. Second, to determine whether the proportion of products featuring each typology category differed across alcohol categories, chi‐squared tests with pairwise *z*‐tests (Bonferroni adjusted alpha (*α* = 0.003)) were conducted for each claim type.

## 3 Results

In total, 5982 products were included in these analyses. Wine represented the largest product category in the dataset (59%), followed by spirits (21%), beer (10%), premix (9%) and cider (2%).

As shown in Table 1, eight types of ‘better for you’ claims were identified: nutrient content, energy content, absence of ingredient, low alcohol, fruit/botanical references, natural, processing and vegan. One‐third of the assessed products (*n* = 2080) displayed at least one type of ‘better for you’ claim, with the cider (91%) and premix (75%) categories containing the highest proportion of products featuring a claim. While the wine and spirits categories contained a relatively low proportion of products featuring at least one claim (26% and 35% respectively), these two categories contained a sizeable number of products featuring claims (*n* = 916 and *n* = 436 respectively).

**TABLE 1:** Typology of text‐based claims identified on alcohol product packaging.

‘Better for you’ claims		
Category	Description	Examples
Nutrient content	Any reference to sugar or carbohydrates	Low sugar Zero carb Lower carb < 2 g sugar
Energy content	Any reference to energy, kilojoules or calories	80 cal Low calorie 52 cal per 100 mL
Absence of ingredient	Any reference to the absence or omission of an ingredient	No added colours No additives No artificial flavours No sulphites Gluten free
Low alcohol	Any reference to a product being low/lower in alcohol content	Low in alcohol Lighter in alcohol
Fruit/botanical references	References to fruit or botanical ingredients/flavours on pack or in a product name (excludes ingredients list)	Watermelon vodka Strawberry flavoured Mango seltzer Gin and peach soda
Natural	Any reference to the words ‘natural’ or ‘pure’	Natural flavours Natural ingredients Pure Natural
Processing	Any reference to a health‐related production process	Organic Freshly crushed Biodynamic
Vegan	Any reference to the word ‘vegan’ or lack of animal products/ingredients	No animal products Vegan friendly Vegan

Analyses by the claim types (See Table 2) found that the most common claims across the sample were references to vegan (13%), followed by fruit/botanical references (12%) and then natural (10%). Claims referring to the absence of an ingredient, nutrient content, and processing practices were present on 7%, 5% and 4% of products respectively, while only 3% of products featured an energy content claim. Notably, low alcohol claims were rare (1%).

**TABLE 2:** Prevalence of ‘better for you’ claims on alcohol products.

		Alcohol category				
	Total, *N* = 5982	Wine, *n* = 3506 (59%)	Spirits, *n* = 1248 (21%)	Beer, *n* = 592 (10%)	Premix, *n* = 538 (9%)	Cider, *n* = 98 (2%)
	%	%	%	%	%	%
Displayed any type of claim	35	26^d^	35^a^	40^a^	75^c^	91^b^
Nutrient content	5	0^d^	1^b^	13^a^	36^c^	15^a^
Energy	3	1^b,c^	< 0.5^c^	7^a^	23^d^	2^a,b^
Absence of ingredient	7	3^c^	7^b^	20^a^	22^a^	21^a^
Fruit/botanical references	12	2^e^	19^c^	5^a^	57^d^	84^b^
Low alcohol	1	2^a^	< 0.5^b^	1^a^	1^a,b^	0^a,b^
Natural	10	5^c^	13^a^	10^a,b^	30^d^	3^b,c^
Processing	4	3^a,b^	2^a,b^	1^b^	16^c^	4
Vegan	13	18^b^	2^d^	6^a^	12^c^	10^a,b,c^

*Note:* Proportions with the same superscript letter in each row did not significantly differ from each other at a Bonferroni adjusted alpha level of 0.003.

Further analysis across alcohol categories and claim types found that vegan claims were most frequently observed on wine products (18%) and references to fruit/botanical references were more prevalent on cider (84%) and premix products (57%) compared to all other alcohol categories (ranges 2%–19%). Natural claims were most frequently observed on premix products (30%) while absence of ingredient claims were common on premix, cider and beer products (22%, 21% and 20% respectively). Processing claims were most often observed on cider products (16%). Although nutrient content and energy content claims appeared infrequently across most product categories, they were significantly more prevalent on premix, present on 36% and 23% of products respectively.

As shown in Table 3, the median number of claims per product was 0, ranging from a median of 0 for wine, spirits, and beer products to a median of 1 for premix and cider. Among products displaying at least one claim, the median number of claims per product was 1 and was highest for premix and beer products at 2 claims per product. Overall, 722 products (12% of the sample) displayed two or more claims, with the maximum number of claims on any single product being 9, which was observed in the spirits category. Analysis by alcohol category found that spirits (7%) and wine (6%) products rarely featured multiple claims, while premix (44%), cider (44%) and beer (24%) products were found to display two or more claims more frequently. Notably, premix beverages were particularly likely to display a high number of claims, with 24% (*n* = 130) featuring four or more claims compared to 3% (*n* = 4) of ciders and 2% (*n* = 13) of beers.

**TABLE 3:** Prevalence of products overall and by alcohol category displaying 0 through to 9 claims.

Number of claims on product	Total, *N* = 5982	Alcohol category				
		Wine, *n* = 3506 (59%)	Spirits, *n* = 1248 (21%)	Beer, *n* = 592 (10%)	Premix, *n* = 538 (9%)	Cider, *n* = 98 (2%)
	*N* (%)	*n* (%)	*n* (%)	*n* (%)	*n* (%)	*n* (%)
0	3902 (65)	2590 (74)	812 (65)	356 (60)	135 (25)	9 (9)
1	1358 (23)	706 (20)	347 (28)	95 (16)	164 (31)	46 (47)
2	362 (6)	123 (4)	58 (5)	89 (15)	67 (13)	25 (26)
3	177 (3)	69 (2)	13 (1)	39 (7)	42 (8)	14 (14)
4	105 (2)	13 (< 0.5)	14 (1)	7 (1)	70 (13)	1 (1)
5	46 (1)	5 (< 0.5)	2 (< 0.5)	5 (1)	31 (6)	3 (3)
6	29 (< 0.5)	0	0	1 (< 0.5)	28 (5)	0
7	2 (< 0.5)	0	1 (< 0.5)	0	1 (< 0.5)	0
8	0	0	0	0	0	0
9	1 (< 0.5)	0	1 (< 0.5)	0	0	0
Median: All products	0	0	0	0	1	1
Median: Products with ≥ 1 claim	1	1	1	2	2	1

## 4 Discussion

A key contribution of this study was the development of a typology of the types of text‐based ‘better for you’ claims used on alcoholic products. This adds to the literature by providing a systematic overview of the various ways through which alcohol companies may attempt to signal to consumers that a product is ‘better for you’. The application of a consistent classification system has the potential to aid researchers and policymakers in monitoring the use of claims over time, which is particularly important given the apparent growth of the ‘better for you’ market globally [24]. An additional benefit of the typology is the inclusion of a broader set of claims than previously analysed on alcohol products (e.g., fruit/botanical references). The breadth of claims identified in this study highlights the novel ways in which companies are portraying products as ‘better for you’ and, in some cases, adapting claims from the food industry to alcohol products (e.g., gluten‐free).

The present study found that 2080 products (35% of the sample) featured at least one claim and that 722 products (12%) displayed multiple claims that could suggest to consumers that the products bearing the claims are ‘better for you’ than those without them. The substantial number of products across all alcohol categories featuring at least one claim highlights the pervasiveness of ‘better for you’ marketing in the Australian alcohol market and the extent to which consumers are being exposed to such messaging. This trend warrants further attention from researchers and regulators because of the potential to distract consumers from the overall health harms associated with alcohol use [12, 13]. Claims that focus on low sugar content, for example, can distract from the fact that, as well as being a carcinogen [15], ethanol is energy‐dense, typically providing the largest source of calories from alcoholic beverages [33].

A novel finding of the analysis was that the use of multiple claims on individual packages occurred more frequently on premix and cider than on other product categories. This observation is concerning as these products are typically consumed by younger and female drinkers [34] who tend to be more health‐conscious than older, male drinkers [35, 36]. As a result, these groups are potentially more susceptible to being influenced by claims about a product being ‘healthier’ as well as alcohol‐related marketing claims in general [37, 38, 39]. Over the last decade, drinking rates in younger age groups have been in decline [4, 5, 6], while female consumption has been on the rise [40]. The high prevalence of claims on products that are popular among these groups suggests that the alcohol industry may be trying to take advantage of the interest in ‘healthier’ products to halt or reverse declines in youth alcohol consumption and encourage alcohol use among females in particular. Public health policy needs to place particular emphasis on how products are marketed to protect the health gains generated by reduced alcohol consumption in younger age groups [4, 41] and prevent further increases in consumption rates among females.

The results of the present study suggest that the use of fruit terms on alcohol packaging warrants further attention. Existing research in the food context has found that references to fruit on product packaging can increase the perceived healthfulness of unhealthy products [42, 43]. For example, it has been shown that consumers perceive products labelled as containing ‘fruit sugar’ to be healthier than those labelled with ‘sugar’, even when both products contain identical nutritional profiles [43]. Additionally, research from the vaping context has found that products promoted as being fruit‐flavoured are more appealing to younger consumers and are perceived as less harmful to health than unflavoured products [29, 30]. While there is little evidence as to whether similar effects occur on alcohol products, the potential for such claims to reduce perceived harm raises concerns given the serious health risks associated with even low levels of alcohol consumption [14, 15]. Furthermore, alcohol products sold in Australia are not required to provide an ingredient list [22], meaning that the use of fruit flavours has greater potential to mislead, as consumers have no way of determining whether the product contains real fruit. The prevalent use of fruit flavours in the premix and cider categories requires ongoing attention from researchers and policy makers, due to the known appeal of these product categories among younger age groups [44].

From a regulatory perspective, several of the identified claims (e.g., natural) do not fall within the specific remit of the FSANZ Code relevant to the labelling of alcohol products, but rather are subject to the general provisions in Australian Consumer Law that require claims not to be misleading or deceptive [22, 45]. In both alcohol and unhealthy food contexts, vague claims such as ‘natural’ and ‘no artificial sweeteners’ have been found to mislead consumers and result in less healthy product choices [46, 47]. One reason for this is that such claims often lack formal definitions, meaning they can be interpreted in a variety of ways by consumers, including in ways that do not reflect the actual content of the product [48]. The use of this terminology is deserving of further regulatory attention as these claims can potentially be used by industry as a loophole to convey that some types of alcohol are ‘better for you’.

The results of the present study align with growing international interest in this area around minimising the use of claims that give the (mis)perception that alcoholic products can be healthy. For example, the Codex Alimentarius Commission (the international food standards agency that has previously issued global guidance for nutrition labelling of food) is progressing work on alcohol labelling, including the assessment of options to reduce the use of claims on alcohol specifically [49]. National and international alcohol policymakers could also learn from the tobacco control context where the Framework Convention on Tobacco Control required states parties to restrict the use of descriptors such as ‘lite’ and ‘mild’ on tobacco packages, to ensure that consumers were not misled into believing some forms of smoking are less harmful than others given the overall harms of the product [50, 51]. Considering that WHO guidelines on alcohol state that no level of alcohol consumption is considered safe [52], a similar argument could be made in the alcohol context about the need to regulate and remove ‘better for you’ claims that create the impression that some alcohol products can be healthy for you.

### 4.1 Study Limitations

The data set used for this study only included products collected from three alcohol stores in Sydney. While all stores were major retailers, this sample may not be representative of the entire alcohol supply in Australia. This study also only focused on ‘better for you’ messages communicated through text‐based labelling and did not include other forms of on‐pack advertising that also attempt to convey healthiness, such as the use of colour and visual elements of the packaging. Furthermore, while several of the identified claims were defined as ‘better for you’, this analysis could not determine whether these claims influence consumer attitudes and behaviour. For example, while fruit flavour claims have been shown to increase the perceived healthiness of food products [43] and decrease the perceived risk associated with e‐cigarettes [29, 30], it is unclear whether this is also the case for alcohol products. As such, the inclusion of these claims in the typology may act to overestimate the prevalence of claims in the market if such claims are not interpreted by consumers as indicating products are ‘better for you’. Future research should examine whether and how the various types of claims included in the developed typology influence consumers' alcohol‐related perceptions, purchasing, and consumption.

## 5 Conclusion

The results of this study indicate that ‘better for you’ claims are displayed on almost one‐third of alcohol products on the Australian alcohol market, with markedly higher prevalence in product categories that are popular among young people. The widespread use of claims is concerning as the promotion of alcoholic beverages as ‘better for you’ has the potential to mislead consumers about the risks associated with alcohol use. To protect the public, there is a need for further action by researchers and policymakers to develop regulation that limits the use of the claims that can be made in respect of alcohol products.

## Author Contributions

Conceptualisation: Asad Yusoff, Simone Pettigrew and Alexandra Jones. Data collection: Asad Yusoff and Bella Sträuli. Methodology: Asad Yusoff, Alexandra Jones, Simone Pettigrew and Bella Sträuli. Data analysis: Asad Yusoff. Writing – Original draft: Asad Yusoff. Writing – Review and editing: Asad Yusoff, Alexandra Jones, Simone Pettigrew, Bella Sträuli, Paula O'Brien, Jacqueline Bowden and Mark Petticrew. Funding acquisition: Simone Pettigrew, Alexandra Jones, Paula O'Brien, Jacqueline Bowden and Mark Petticrew.

## Funding

This study was funded by the National Health and Medical Research Council Ideas Grant #2021186.

## Conflicts of Interest

The authors declare no conflicts of interest.

## Data Availability

The data that support the findings of this study are available from the corresponding author upon reasonable request.
